# The Phylogeny and Pathogenesis of *Sacbrood Virus* (SBV) Infection in European Honey Bees, *Apis mellifera*

**DOI:** 10.3390/v11010061

**Published:** 2019-01-14

**Authors:** Jianghong Li, Tingyun Wang, Jay D. Evans, Robyn Rose, Yazhou Zhao, Zhiguo Li, Jilian Li, Shaokang Huang, Matthew Heerman, Cristina Rodríguez-García, Olubukola Banmeke, J. Rodney Brister, Eneida L. Hatcher, Lianfei Cao, Michele Hamilton, Yanping Chen

**Affiliations:** 1 USDA-ARS Bee Research Laboratory, USDA-ARS, Bldg. 306, BARC-East, Beltsville, MD 20705, USA; leejh6972@126.com (J.L.); Jay.evans@ars.usda.gov (J.D.E.); zhaoyazhou@caas.cn (Y.Z.); zhiguo.li@fafu.edu.cn (Z.L.); Matthew.Heerman@ARS.USDA.GOV (M.H.); cristinarodriguez.crg@gmail.com (C.R.-G.); olubukola.banmeke@bison.howard.edu (O.B.); lianfeicaocn@gmail.com (L.C.); Michele.hamilton@ars.usda.gov (M.H.); 2 College of Bee Science, Fujian Agriculture and Forestry University, Fuzhou 350002, China; wangtingyun3@126.com (T.W.); skhuang@fafu.edu.cn (S.H.); 3 USDA APHIS, National Program Manager for Honey Bee Health, Riverdale, MD 20737, USA; Robyn.I.Rose@aphis.usda.gov; 4 Institute of Apicultural Research, Chinese Academy of Agriculture Sciences, Beijing 100081, China; bumblebeeljl@hotmail.com; 5 Laboratorio de Patología Apícola, Centro de Investigación Apícola y Agroambiental, IRIAF, Consejería de Agricultura de la Junta de Comunidades de Castilla-La Mancha, 19180 Marchamalo, Spain; 6 National Center for Biotechnology Information, National Institutes of Health, Bethesda, MD 20894, USA; jamesbr@ncbi.nlm.nih.gov (J.R.B.); eneida.hatcher@nih.gov (E.L.H.); 7 Institute of Animal Science and Veterinary Medicine, Zhejiang Academy of Agricultural Sciences, Hangzhou 310021, China

**Keywords:** honey bee, *Sacbrood virus* (SBV), genome, genetic variation, phylogeny, cold stress, pathogenicity

## Abstract

RNA viruses that contain single-stranded RNA genomes of positive sense make up the largest group of pathogens infecting honey bees. *Sacbrood virus* (SBV) is one of the most widely distributed honey bee viruses and infects the larvae of honey bees, resulting in failure to pupate and death. Among all of the viruses infecting honey bees, SBV has the greatest number of complete genomes isolated from both European honey bees *Apis mellifera* and Asian honey bees *A. cerana* worldwide. To enhance our understanding of the evolution and pathogenicity of SBV, in this study, we present the first report of whole genome sequences of two U.S. strains of SBV. The complete genome sequences of the two U.S. SBV strains were deposited in GenBank under accession numbers: MG545286.1 and MG545287.1. Both SBV strains show the typical genomic features of the *Iflaviridae* family. The phylogenetic analysis of the single polyprotein coding region of the U.S. strains, and other GenBank SBV submissions revealed that SBV strains split into two distinct lineages, possibly reflecting host affiliation. The phylogenetic analysis based on the 5′UTR revealed a monophyletic clade with the deep parts of the tree occupied by SBV strains from both *A. cerane* and *A. mellifera*, and the tips of branches of the tree occupied by SBV strains from *A. mellifera*. The study of the cold stress on the pathogenesis of the SBV infection showed that cold stress could have profound effects on sacbrood disease severity manifested by increased mortality of infected larvae. This result suggests that the high prevalence of sacbrood disease in early spring may be due to the fluctuating temperatures during the season. This study will contribute to a better understanding of the evolution and pathogenesis of SBV infection in honey bees, and have important epidemiological relevance.

## 1. Introduction

Honey bees are the most important pollinator of food crops and flowering plants, and are therefore indispensable for agricultural food production and ecological balance [1,2,3]. While the demand for pollination service for crop cultivation has tripled over the past decades [2,4], the honey bee population has been declining at an alarming rate in the United States and many European countries [4,5]. During the winter of 2006–2007, honey bee Colony Collapse Disorder (CCD), a mysterious malady that is characterized by the abrupt disappearance of all adult workers in a hive, wiped out an estimated 10 million beehives in the U.S. [6]. Since then, honey bee populations have continued to dwindle each year with honey bee colony losses averaging more than 30% annually in the U.S. [7]. Nevertheless, the precise reasons for the high level of bee losses have not yet been completely understood. A number of different factors including parasites and disease, genetics, poor nutrition, and pesticide exposure have been suggested to play roles in the decline of honey bee populations [8,9]. Of the factors adversely affecting bee health, viruses have often been linked to colony losses; including the losses caused by CCD, and have been a subject of major bee health concern [10,11,12].

Honey bee sacbrood disease is caused by the *Sacbrood virus* (SBV) which belongs to a family of viruses termed *Iflaviridae* and is the first bee virus to be described [13]. Since its first identification in the U.S. in 1913 [13], infections of SBV have been found in every part of the world where beekeeping practices are present [14,15]. Sacbrood derives its name from the saclike appearance of the diseased larvae. SBV mainly infects early stage larvae [15,16]. The young larvae become infected with the virus by ingesting the glandular secretions of SBV-infected nurse bees. They are unable to shed their leathery endocuticle, under which a large amount of ecdysial fluid containing millions of SBV particles accumulates between the body of a diseased larva and its unshed cuticle. As a result, they form a sac-like appearance that is characteristic of this disease. The infected brood fail to pupate and larvae usually die at the last larval (prepupal) stage. The viruses may remain viable in dead larvae, honey, or pollen for up to four weeks. While the larval stage is the most susceptible to SBV infection, SBV can also affect adult workers by having an adverse effect on their longevity. The viruses are frequently present as a latent infection in seemingly healthy adult bees, and accumulate in the hypopharyngeal gland of adult workers which contribute to the spread of the virus by feeding larvae with the virus contaminated brood food of their secretions [17,18]. SBV infection can be further spread from one hive to another through robbing and drifting of the virus-infected colonies. Sacbrood disease occurs from early spring through late fall but is most commonly seen in the early spring [19,20,21].

Stressors that affect bee health and colony productivity can have direct effects on sacbrood disease pathogenicity [22]. Changing temperature, which is more often a stressor of concern, could influence the prevalence of SBV infection in honey bees. The seasonal variation of SBV infection has been documented, and SBV is prominently found in Spring when temperature tends to fluctuate [21]. *Varroa destructor* is an ectoparasitic mite that parasitizes the honey bees and has been catastrophic for the beekeeping industry since it jumped host from *A. cerana* to *A. mellifera* [23,24]. *Varroa* weakens the honey bees by sucking on the blood of adult bees, brood stage, and also serves as a vector for a variety of honey bee viruses [25]. More recent studies observed that the *Varroa* magnifies the impact of *Deformed wing virus* (DWV), one of the most prevalent viruses affecting honey bees [15,26], and the DWV-*Varroa* association has decimated millions of honey bees worldwide [24]. Previous reports showed that *Varroa* also plays a role in SBV transmission and the prevalence of SBV in honey bee colonies was found to be positively correlated with the level of *Varroa* infestation [21]. The synergistic negative effects of *Varroa* mite infestation and SBV infection have detrimental effects on bee health.

Among the pathogenic groups, RNA viruses account for 38% of all emerging and re-emerging disease infections due to their exceptionally high mutation rates [27]. All RNA viruses do not include a built-in exonucleolytic proofreading step in their replication, thereby resulting in generations of many genomic variants within a viral population. According to the available sequence record in GenBank, SBV has the hightest record of whole genome sequences among all the viruses infecting honey bees. SBV infects both the European honey bee, *Apis mellifera*, and the Asian honey bee, *A. cerana* [18,28,29,30,31]. While SBV disease has been reported to affect about 15% of *A. mellifera* and could have seriously detrimental effects on colony health [32], this virus is the most dangerous pathogen of *A. cerana* and has been linked to the collapse of Asian honey bee colonies. Historically, the catastrophic outbreak of SBV disease killed 100% of *A. cerana* colonies in Thailand in 1976, 95% of *A. cerana* colonies in India in 1978, and completely destroyed the Korean apiculture industry in 2010 [30,33,34,35]. The severe losses of *A. cerana* populations across Asia due to SBV disease were caused by a variety of strains of the virus reflective of their geographic regions of isolation; namely Chinese SBV, Korean SBV, Thai SBV, and so on [33,36,37,38,39,40]. Recent studies have shown that the *A. cerana* strain of SBV could cause infection in *A. mellifera* experimentally [41,42], indicating that SBV is capable of crossing the species barrier to establish infection and posing a new threat to the European honey bee, *A. mellifera*. Therefore, it is critically important to improve our understanding of the evolution, pathogenesis and geographical range of SBV to enhance our preparedness and response for coping with novel emerging disease threats. 

In the present study, we report the whole genome sequences of two U.S. strains of SBV and the phylogenetic analysis of SBV strains around the world. The effects of cold temperatures on SBV pathogenic infection in bee larvae were also studied. To our knowledge, this is the first report of the complete genome sequences and phylogenetic analysis of this potentially damaging virus from the U.S.

## 2. Materials and Methods

### 2.1. Ethics Statement

No specific permits were required for the studies described. Observations were made in the United States Department of Agriculture (USDA)-Agricultural Research Service (ARS) Bee Research Laboratory apiaries, Beltsville, Maryland, USA. The apiaries are the property of the USDA-ARS and are not privately-owned or protected in any way. Studies involved the European honey bee (*Apis mellifera*) which is neither an endangered nor protected species.

### 2.2. Bee Sample Collection and SBV Inoculum Preparation

SBV-infected bees (*A. mellifera ligustica*) were collected from colonies maintained in the apiaries of the USDA-ARS Bee Research Laboratory in Beltsville, MD. Honey bee colonies were screened monthly to determine the incidence and prevalence of virus infections including *Acute bee paralysis virus* (ABPV), *Black queen cell virus* (BQCV), *Chronic bee paralysis virus* (CBPV), *Deformed wing virus* (DWV), *Israeli acute paralysis virus* (IAPV), *Kashmir bee virus* (KBV) and SBV using Reverse Transcription-Polymerase Chain Reaction (RT-PCR), as described previously [43]. The information regarding primer sequences and size of PCR products are shown in Supplementary Table S1. SBV-infected larvae and adults were sampled from the bee colonies with clinical signs of the sacbrood disease, and that were confirmed only with SBV infection by molecular method [44]. SBV-infected larvae were collected with fine insect forceps and transferred into 1.5 mL Eppendorf tubes individually. Adult bees were collected from the bee frames by gently scooping worker bees into 50-mL conical tubes. Thirty larvae and adults were collected per colony, and all samples were stored in a –80 °C freezer for subsequent RNA extraction.

The SBV inoculum was prepared from the SBV-infected larvae collected. Briefly, SBV-infected larvae in 1.5 mL Eppendorf tubes were homogenized individually in 500 µL sterile phosphate-buffered solution (PBS) (137 mM NaCl, 2.7 mM KCl, 10 mM Na2HPO4, 1.8 mM KH2PO4, pH 7.4) with a sterile plastic pestle. The homogenized solution was centrifuged at 14,000 g at 4 °C for 10 min, and the supernatant was further passed through a 0.45 µm filter and 0.22 µm cell filter successively. 100 µL of the viral solution was used for RNA extraction and RT-PCR assay using SBV specific primers for the confirmation of the SBV infection and the exclusion of the presence of other viruses [44]. The rest of the viral solution was used for preparing the inoculum for subsequent virus infection assay to determine the effects of cold stress on the pathogenesis of the SBV infection in *A. mellifera* larvae.

### 2.3. RNA Extraction

The larvae and adult bee samples were taken out from the −80 °C freezer and ground in a 1.5 mL Eppendorf tube with a sterile plastic pestle individually for RNA extraction. The total RNA was extracted using TRIzol, a ready-to-use reagent for the isolation of total RNA (Invitrogen, Carlsbad, CA, USA), following the manufacturer’s instructions. Following precipitation and centrifugation, the resultant RNA pellets were re-suspended in UltraPure DNase/RNase-Free Distilled Water (Invitrogen), with the addition of Ribonuclease Inhibitor (Invitrogen). The quantity and purity of RNA were measured using a NanoDrop 8000 Spectrophotometer (NanoDrop Technologies, Wilmington, DE). The RNA samples were kept in −80 °C until further use.

### 2.4. RT-PCR and RACE

**Figure 1:**
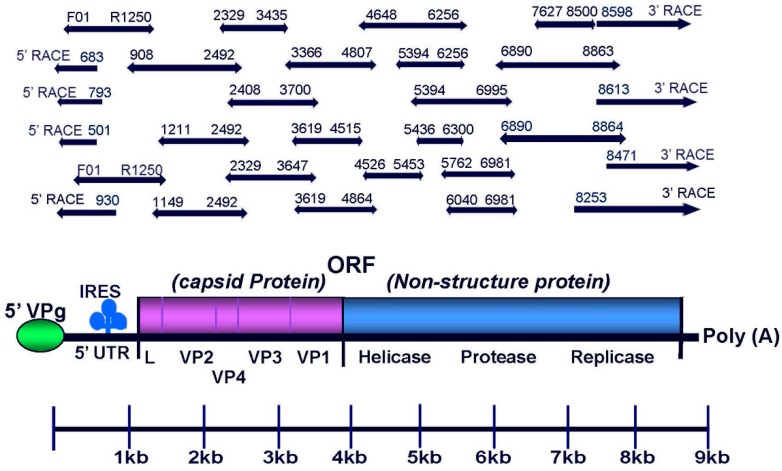
Schematic diagram of the *Sacbrood virus* (SBV) genome organization and overlapping PCR fragments and cDNA ends that span the entire viral genome. Like other members of the *Iflaviridae* family, the genome of SBV is monopartite monocistronic with genes encoding capsid proteins at the 5′end and genes encoding replicase proteins (Hel, Pro, RdRp) at the 3’s end. The full-length SBV genomes were sequenced using a combination of RT-PCR amplification and methods for rapid amplification of 5′ and 3′ cDNA ends (5′ RACE and 3′ RACE).

A series of overlapping fragments spanning the SBV genome were generated by combining primer walking and RT-PCR amplification using the Promega one-step Access RT-PCR system (Madison, WI, USA) following the manufacturer instructions (Figure 1). The sequences of the 5′- and 3′-ends of genome were determined by 5′ Rapid Amplification of cDNA End (RACE) System and 3′ RACE System, respectively (Invitrogen). The primers were designed based on the RefSeq SBV genome (NC_002066.1). The information regarding sequences and genomic positions of primers used to amplify overlapping RT-PCR fragments, 5′-RACE, and 3′-RACE nested PCRs are shown in Table 1. All amplified PCR products were analyzed together with 100 bp DNA ladder for determination of the size of PCR products by electrophoresis using 1% agarose gel. Individual DNA fragments were purified from the agarose gel using the Wizard^®^ PCR Preps DNA Purification System (Promega), and subjected to two directional Sanger sequencing. Overlapping sequences were assembled into a complete SBV genome using SeqMan (DNASTAR, Madison, WI, USA).

### 2.5. Phylogenetic Analysis

To understand the phylogenetic relationship of SBV strains in the U.S. versus worldwide, nucleotide sequences corresponding to the 5′ untranslated region (UTR) and the coding region for the structural and nonstructural polypeptide of the SBV genome were used to create phylogenetic trees. The sequence of *Infectious flacherie virus* (GenBank accession: NC_003781.1) was used as an outgroup to root the trees. The translated polyproteins and 5′ UTRs of the nucleotide sequences of two U.S. strains of SBV obtained in the present study and 52 SBV strains that were retrieved from GenBank.were aligned individually using MUltiple Sequence Comparison by Log-Expectation (MUSCLE) 3.8 [44], implemented in Geneious Prime 2019.0.4 [45]. In order to keep the alignment length constant, 10 of the sequences with incomplete 5′ UTRs were removed from the alignment. The phylogenetic trees were inferred by using the Maximum Likelihood method based on the MrBayes [46,47]. Parameters were set to estimate the polyprotein tree using mixed phylogenetic models, and 5′ UTR tree was estimated with the General Time Reversible (GTR) model with gamma-distributed rate variation across sites and a proportion of invariable sites. For both trees, the first 25% of the samples were discarded (burn-in), and the analysis was allowed to run until convergence was reached (the average standard deviation of split frequencies is < 0.01).

### 2.6. Laboratory Induced Sbv Pathogenic Infection

To obtain age-controlled larvae, bee colonies with active egg laying queens and identified as free of virus infection by RT-PCR assay were selected for the study. For each colony, an egg laying queen along with an empty frame and 2–3 frames full of brood were put into the bottom box of a bee hive colony separated from the upper box by a queen excluder to allow the queen to lay eggs. After six hours, the frame with eggs 3 ± 3 h old was marked and moved to the upper box for incubation in the colony under natural conditions.

The purified SBV inoculum (350 µL) mentioned above was mixed with 1 mL of larval food containing 50% fresh royal jelly, 6% glucose, 6% fructose, and 1% yeast extract [48]. This mixture was used as an inoculum to induce SBV infection in bee larvae. Specifically, the 2nd instar larvae (108 ± 3 h post laying) were grafted from the frame and transferred into a 24-well Corning^®^ Costar cell culture plate containing a mixture of virus inoculum and larval food which was warmed at 34.5 °C for 10 min. A desiccator was used to house the cell culture plates and placed in an incubator at 34.5 °C and 95% relative humidity (RH) for 24 h. After 24 h, the larvae were rinsed in 34.5 °C warmed water twice to remove the SBV inoculum on the body surface and then put into another new 24-well plate containing only warmed larvae food. The larval food was changed every 24 h to provide fresh diet until pupation. 

Eight SBV inoculated larvae were randomly sampled after 24 h incubation and washed in PBS solution to remove contamination from the larvae food. Under a stereomicroscope, hemolymph was collected using a fine glass capillary tube and transferred into an Eppendorf tube. The midguts were separated from the body by using two fine forceps and washed in PBS twice to avoid possible contaminants from hemolymph and other tissues. Both isolated hemolymph and midgut tissues were processed immediately for RNA isolation. The presence of SBV in the hemolymph and the spread of the SBV infection from hemolymph to midgut of the SBV-inoculated larvae were examined by RT-PCR assay.

### 2.7. Cold Stress on Survivorship of SBV-Infected Larvae

A pilot study was conducted to determine the effect of cold stress on the larval development and metamorphosis. Bee colonies without detectable virus infection and used for receiving SBV inoculation above were used in the study. The fifth instar larvae (192 h after egg laying) that stopped eating were removed from the brood frame and laid on three layers of filter paper inside of a petri dish (100 × 15 mm). Three Petri dishes (*n* = 3 × 10 larvae) per group were incubated at one of the five different temperatures: 15 °C, 20 °C, 28 °C, 33 °C, or 35 °C. Mortality of larvae was recorded every day. Larvae were classified as dead when their color changes to brownish. The number of larvae finishing its metamorphosis development under different temperatures and reaching to the pupal stage was also recorded.

To explore the effect of cold stress on SBV’s pathogenicity, 2nd instar SBV-infected larvae and 5th instar SBV-infected larvae were subjected to a cold challenge by incubation at 20 °C for six hours. Afterward, the cell culture plates were put back into the incubator set at 34.5 °C. The number of dead larvae was recorded every day and the larvae mortality before pupation was calculated. There were four groups: I—negative control without SBV infection or cold challenge, II—group with SBV infection but no cold challenge, III—group with SBV infection and cold challenged at the 2nd instar larvae stage, and IV—group with SBV infection and cold challenged at the 5th instar larvae stage. Each group was replicated in three 24-well culture plates (*n* = 24 × 3). All data were expressed as the mean ± SEM. One way ANOVA in SPSS (PASW Statistics 18, SPSS Inc.) was used for analyzing the difference in larvae mortality among the different groups. A post hoc multiple comparison was performed using the method of Tukey’s-b when the equal variance was assumed or Dunnett’s T3 when the equal variance was not assumed. In all cases, a *p*-value of ≤ 0.05 was taken to indicate statistical significance. Because the data are binomial in nature, statistical analysis was based on a generalized linear mixed model (because random effects were included, using the logit link and R software with the lme4 package [49]. The combination of lowest Akaike information criterion (AIC) and main effects retention (i.e., preserve main effects in the model even if not significant as long as higher order terms involving these main effects were significant) were used to select a model that captured the important features of the data.

## 3. Results

### 3.1. Complete Genome Sequences of U.S. Strains of SBV

Two full-length SBV genomes derived from U.S. bee samples were sequenced. The complete genome sequences of the two U.S. SBV strains were deposited in GenBank under accession numbers: MG545286.1 and MG545287.1. Both SBV strains show the typical genomic features of the *Iflaviridae* family: positive-sense, single-stranded RNA genome, approximately 8861 nt long, and contains a single open reading frame (ORF). The ORF encodes a polyprotein of 1942 amino acids flanked by approximately 188 nt of 5′-UTR and approximately 93 nt of 3′-UTR which terminates in a poly(A) tail. The C-terminal portion of the polyprotein possesses consensus sequences in the order of helicase, protease, then RNA-dependent RNA polymerase. The N-terminal portion of the polyprotein shows homology with the coat proteins (CPs) of other iflaviruses in the order VP2–VP4–VP3–VP1 (Figure 1).

### 3.2. Genetic Relationship of SBV Strains Worldwide

**Figure 2:**
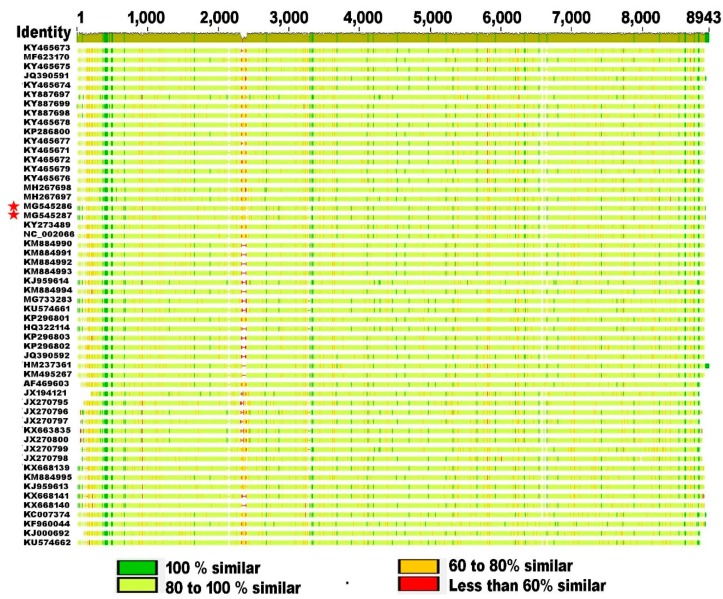
Alignment of SBV genomes. The 52 SBV genomes that are available from the NCBI Viral Genomes Resource [PMID: 25428358], including the RefSeq genome of SBV NC_002066.1, were aligned using MUSCLE. The columns are numbered by the alignment coordinates, and the identity graph in the top panel is colored by conservation, with height indicating coverage. The polyprotein spans from approximately position 200 to 8800. The USA isolates described in this manuscript are highlighted by red stars. The color boxes at the bottom panel shows the individual aligned sequences with positions colored by the level of conservation.

To date, there are 52 strains of SBV that have been isolated from *A. mellifera* worldwide and *A. cerana* in southern Asia, and their whole genomic sequence data have been reported and deposited in the NCBI Viral Genomes Resource Comparison of SBV stains worldwide at the genome level shows a significant genetic divergence among different strains with level of nucleotide variation ranging from 3% to 11% at the genome level, providing evidence of quasi-species dynamics in SBV populations. The whole genome squence alignment illustrats the conservation, differences, and gaps of genomic regions and that the polymorphisms tend to bemore frequently in 5′ UTR compared to the protein coding region and 3′ UTR (Figure 2).

**Figure 3:**
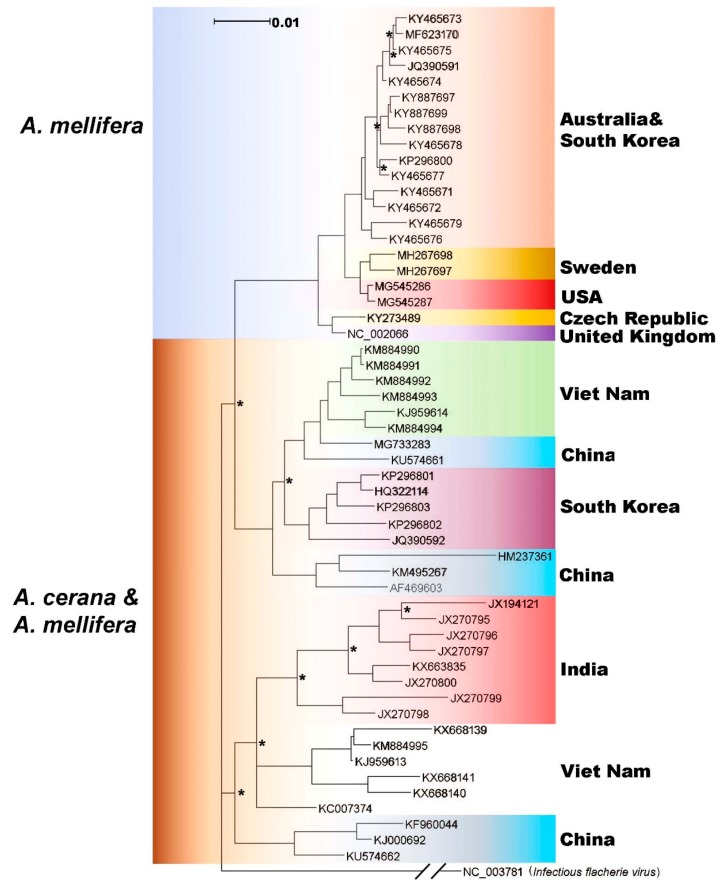
Phylogenetic tree of tree of the translated polyprotein. SBV sequences were retrieved from GenBank, and the translated polyproteins were aligned using MUSCLE. The phylogenetic tree was inferred by using MrBayes, and the analysis parameters were set to estimate relationships using mixed phylogenetic models. *Infectious flacherie virus* (IFV, GenBank accession: NC_003781.1) was used as an outgroup to root the trees. Tree branches are colored by isolation host on the left, and country of isolation on the right. In some cases, isolates from multiple countries were intermingled, and are shown using the same color (e.g., Australia and South Korea). Nodes that are not supported with clade credibility values ≥90 are marked with *.

The phylogenetic tree based on the polyprotein was divided into three main branches. One branch consisted of sequences isolated from only *A. mellifera*, and the other branches contain a mix of sequences from both *A. mellifera* and *A. cerana*. Within the monophyletic group of SBV strains from *A. mellifera*, the U.S. strains of SBV are most similar to SBV isolated from honey bees in Sweden. From the data available at this time, it cannot be determined whether these sequences group together because they are becoming more adapted to *A. mellifera*, or because geographic distance has isolated the sequences, allowing diversification from the rest of the tree. Sequences on the other two branches of the tree were isolated from countries in Asia, including Vietnam, South Korea, India, and China. Several of these countries appear to have multiple linneages of SBV circulating within their bees. Also, on these two branches, sequences from both *A. mellifera* and *A. cerana* and found near each other, indicating that SBV is most likely able to jump between the hosts (Figure 3).

**Figure 4:**
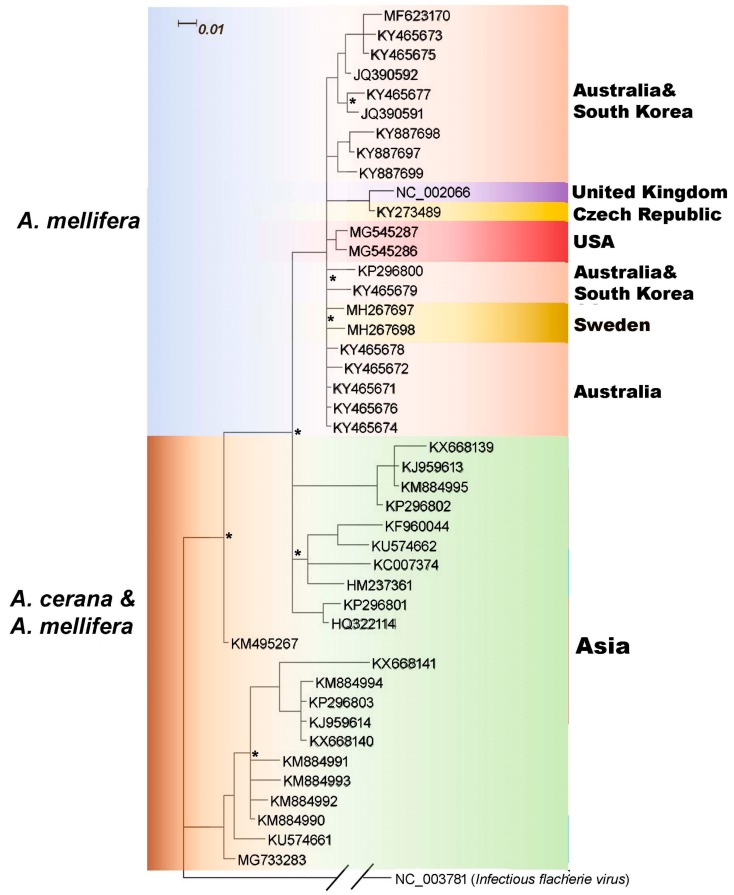
Phylogenetic tree of the 5′ untranslated region (5′ UTR) SBV sequences were retrieved from GenBank, and were trimmed to the 5′ UTRs. The phylogenetic tree was inferred by using MrBayes, and the analysis parameters were set to estimate relationships using the GTR model with gamma-distributed rate variation across sites and a proportion of invariable sites. Infectious flacherie virus (IFV, GenBank accession: NC_003781.1) was used as an outgroup to root the trees. Tree branches are colored by isolation host on the left, and country of isolation on the right. In some cases, isolates from multiple countries were intermingled, and are shown using the same color (e.g., Australia and South Korea). Nodes that are not supported with clade credibility values ≥90 are marked with *.

The 5′ UTR of SBV genome harbors an Internal Ribosome Entry Site (IRES), an RNA element that allows for translation initiation in a cap-independent manner, that directs viral replication. Therefore, 5′ UTR is fundamentally important for virus identification and evolutionary studies. The phylogenetic analysis based on 5′UTR generally showed the similar patterns to the polyprotein phylogeny; European, Australian, some South Korean, and the USA isolates, which were all isolated from *A. mellifera*, form a monophyletic branch. 5′ UTR sequences from other branches on the tree do not discriminate linneages from the Asian countries or hosts they were isolated from by the methods used here. (Figure 4).

### 3.3. Effect of Cold Stress on Mortality Of SBV-Infected Larvae

**Figure 5:**
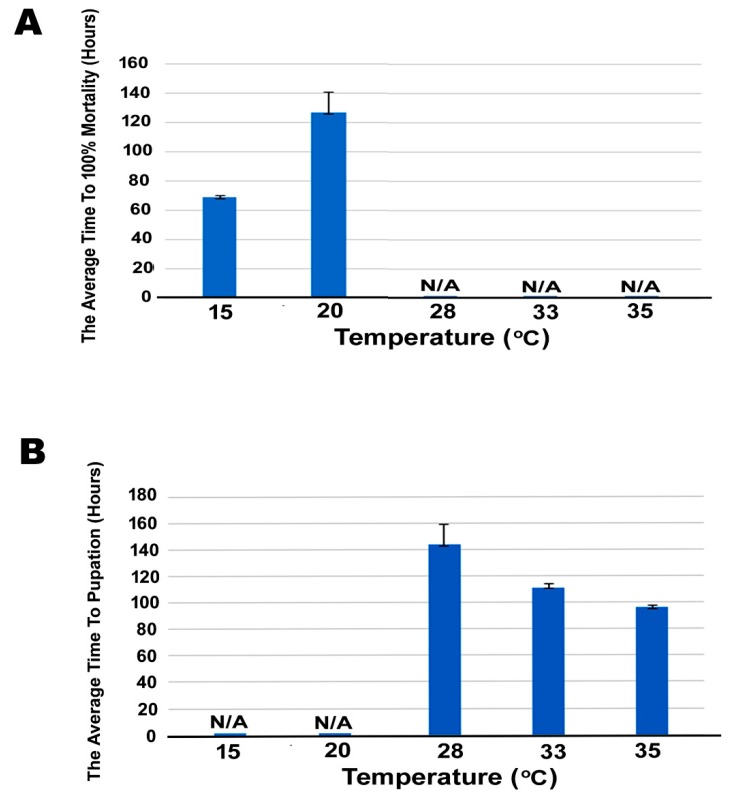
The effect of low temperatures on larvae development and metamorphosis. (**A**). The average time (h) that larvae reached to 100% mortality at different temperatures (15 °C, 20 °C, 28 °C, 33 °C, or 35 °C). N/A (Not Applicable) (**B**) The average time (h) that 5th instar larvae reached to pupation at different temperatures (15 °C, 20 °C, 28 °C, 33 °C, or 35 °C). N/A (Not Applicable).

The effect of temperatures (15 °C, 20 °C, 28 °C, 33 °C, or 35 °C) on larvae development and metamorphosis showed that the fifth-instar larvae incubated at 15 °C started to die after 24 h of incubation and larvae incubated at 20 °C started to die at about 48 h of incubation. 100% larvae incubated at 15 °C, and 20 °C died before pupation (68.62 ± 1 h, and 127.69 ± 8.31 h, respectively). As a result, 20 °C was chosen for the subsequent cold-challenging experiment to test the effect of cold stress on mortality of SBV-infected larvae. All fifth-instar larvae incubated at 33 °C and 35 °C, which is considered as optimum temperature for growth, reached to the pupal stage in 111.42 ± 1 h and 95.95 ± 1.96 h, respectively. 50% of fifth-instar larvae incubated at 28 °C reached the pupal stage and took an average of 144 ± 15.32 h to complete larval-to-pupae development (Figure 5B).

**Figure 6:**
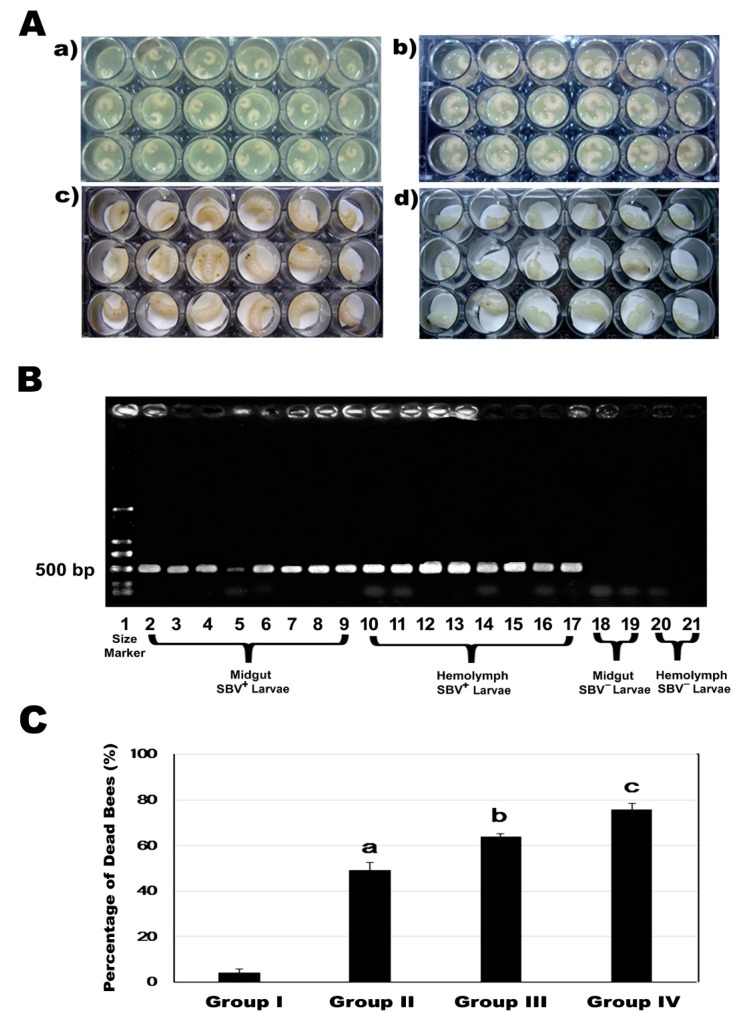
Effect of cold stress on SBV infection. (**A**) SBV infection in vivo. a) 2nd instar larvae (108 ± 3 h post laying), b) 3rd instar larvae, c) 4th instar larvae, and d) 5th instar larvae. (**B**) Detection of SBV by RT-PCR and gel electrophoresis. RNA was extracted from midgut and hemolymph of larvae after 24 h SBV inoculation and examined for the presence of SBV by RT-PCR method and electrophoresis. The number 1 indicates a size marker, numbers 2–9 indicate the midgut tissue of SBV-infected larvae, numbers 10–17 indicate the hemolymph of SBV-infected larvae, numbers 18–19 indicate the midgut tissue of uninfected larvae (negative control), and numbers 20–21 indicate the hemolymph of uninfected larvae (negative control). The size of PCR fragments is indicated on the left of the gel. (**C**) Larvae mortality for the cold challenge and pupation among the four experimental groups. Group I—negative control without SBV infection or cold challenge, Group II—group with SBV infection but without cold challenge, Group III—group with SBV infection and cold challenged at the 2nd instar larvae, and Group IV—group with SBV infection and cold challenged at the 5th instar larvae. Different letters on the top of individual bars indicate significant differences (*p* ≤ 0.05).

RT-PCR assay of the midgut and hemolymph of the larvae inoculated with SBV (Figure 6A) showed that a SBV specific signal could be detected both in the midgut and hemolymph (Figure 6B), indicating that larvae could be infected by consuming brood food containing the SBV particles and that SBV could quickly pass through the midgut protective barrier and reach the hemolymph within 24 h post infection.

Laboratory-induced SBV infection and cold challenge assay showed that cold stress could induce fatal infection by SBV, as illustrated by the increased mortality of SBV-infected larvae exposed to the cold challenge at both 2nd instar (Group III) and 5th instar (Group IV), compared to SBV-infected larvae without the cold challenge (Group II) and negative control of healthy larvae without SBV infection or cold challenge (Group I) (Figure 6C).

The mortality of honey bee larvae in the four groups was significantly different (One way ANOVA, F3, 12=163.580, *p* < 0.001). The next post hoc multiple comparisons showed that SBV infection could lead to fatal infection of bee larvae, 50% of larvae died before pupation in Group II, which is a significant increase in the mortality rate compared to 4% mortality rate in Group I (negative control) (between group I and II, Tukey’s-b, *p* < 0.001). The cold challenge of 20 °C for 6 h on 2nd instar SBV-infected larvae could significantly increase mortality of the larvae, 64% of larvae died before pupation in Group III (between group II and III, Tukey’s-b, *p* = 0.005). Further, a cold challenge of 20 °C for 6 h on 5th instar SBV-infected larvae could lead to an even higher rate of mortality, 76% of larvae died before pupation in Group IV (between group III and IV, Tukey’s-b, *p* = 0.023).

## 4. Discussion

To date, there are no complete genome sequences of the U.S. strains of SBV publicly available. This study aimed to fill the gap by providing such documentation to serve as a background of geographic origin of the U.S. strains to monitor the emergence of new adaptive mutations in the future.

Due to the lack of proofreading function during RNA replication, like other RNA viruses, SBV has a high mutation rate which contributes to the vast diversity of SBV strains circulating in both *A. cerana* and *A. mellifera* populations globally. At present, there are 52 strains of SBV that can be found in the GenBank database. Genome-wide analysis of SBV strains revealed that nucleotide substitutions, insertions, and deletions are spread throughout not only the coding region for the structural and nonstructural polypeptide but also 5′ and 3′ untranslated regions of the virus. Further study of the relationships between genetic variability and virulence of the virus is required.

The phylogenetic tree based on the single polyprotein coding region revealed that SBV strains split into two distinct lineages, reflecting their geographic distribution and possibly host affiliation. Of all the complete genomic sequences of SBV that have been deposited in GenBank, U.S. strains of SBV are most closely related to the UK and Sweden strains of SBV, which may reflect their relatively close geographic proximity and frequent interactions between the locations. SBV infects both *A. cerana* and *A. mellifera*, but is much more destructive to *A. cerana*. In other words, *A. mellifera* seems to be more resistant to this SBV infection. Animal hosts and pathogens have evolved through an arms race of attack and defense strategies [27]. When a host evolves new defenses against a pathogen, the pathogen is forced to adapt its own invade-and-infect strategy to counteract the host’s immune defenses. In response, the host must develop new defense mechanisms to deal with the new attacks, and the rivalry continues and escalates. It was therefore suggested that resistant hosts will impose stronger selection on pathogens than susceptible hosts, which should facilitate and accelerate the evolution of pathogens [28]. Our phylogenetic results may support this suggestion that strong immune response to SBV infection in *A. mellifera* may drive the evolution of the virus.

Another important goal of this study was to investigate the effects of cold stress on the pathogenicity of SBV infection in honey bees. The incidence and prevalence of SBV in honey bees have been notably seasonal. Outbreaks of sacbrood disease often occur from early spring through late fall but are most commonly seen in the early spring. The prevalence of SBV is believed to be positively correlated with the number of susceptible brood in the colonies [13]. During the spring, the new sources of pollen and nectar stimulate brood rearing, providing opportunities for SBV to infect bees and multiply in the colonies. Spring which is characterized by the fluctuations between cold and warm days can cause substantial variations in animal immune functions, therefore affecting an abundance of pathogens [50]. Our study clearly showed that a cold challenge could have profound effects on sacbrood disease severity which was manifested by increased mortality in SBV-infected larvae. This result suggests that spring climate variability is likely a contributing factor to the high prevalence of sacbrood disease in the spring. While molecular mechanisms underlying the effects of cold temperature on SBV pathogenicity remain to be determined, a study in mosquito *Aedes aegypti* revealed that cooler temperatures could destabilize RNAi pathway of the mosquito and increase the susceptibility of the mosquito to virus infections [14]. RNAi plays a pivotal role in the control of viral infection in insects, including honey bees [51,52]. Based upon previous reports and our observation, it is plausible to suggest that cold stress possibly causes the reduction in metabolic rate and immune function including RNAi mechanism, thereby resulting in SBV infections being most severe in infected larvae. Further studies are warranted to examine the effects of cold temperature on RNAi machinery in antiviral defense in honey bees.

Temperature is an important factor affecting insect brood development [53]. A study in mosquitos demonstrated that the larval rearing temperature could affect important developmental parameters of the mosquito, including pupation and emergence time, survival rates, and body size [54]. Honey bees have brood temperature ranges between 32 °C and 36 °C, with a mean of 34.5 °C [55]. Extreme temperatures can have lethal effects on honey bees [56]. Moreover, a study by Wang et al. [56] suggests that prepupae and pupae before eclosion are the most sensitive stages to low-temperature stress. In line with Wang et al.’s findings, our study showed that the 5th instar larvae were more vulnerable to the cold challenge than the 2nd instar larvae as demonstrated by the fact that the 5th instar SBV-infected larvae had a significantly higher mortality rate than the 2nd instar SBV-infected larvae after the cold challenge. The difference in susceptibility to the cold challenge between early and late instar larvae could be attributed to many physiological changes during larval-pupal metamorphosis.

In summary, the genome sequence and phylogenetic analysis reported here will enhance our understanding of the evolutionary characteristics and molecular epidemiology of SBV in different parts of the world. An improved understanding of the effect of cold temperature on SBV disease pathogenicity and brood development and survivorship gained from this study can help improve honey bee management strategies.

## Figures and Tables

**Table 1:** Primers used in the RT-PCR, amplifying the overlapped SBV genomic fragments for sequencing.

Primer Name	Primer Sequences (5′ to 3′)
F01	TACGAATCGTGATTCGATTCATT
F908	ACGCTAAGTGTGCGCCTAAT
F1149	GATATCATCGCGCCTTTGTT
F1211	GGAAGTTTGCTAGTATTTACGTG
F2329	TGGAGGTAAGGGACAACCTG
F2408	TGGACACTGGTGCTAAAGAAGATG
F3083	GAAGCTGGGGATGATTTTGA
F3366	CGCTATAGGTGGCATGCAGA
F3619	GGCATGGATTGATCGAAGTT
F4526	CCGGAAATGGCTCATTTAGA
F4648	TACCGTAGGAGAGATGTGTTATTGT
F5394	TCGAGAAGTGGTTCAGTGCC
F5436	AAGTAGTCCAGTGCCCGATG
F5762	TCGGATGGTGAAGATGATGA
F6040	CCAGCGGTACAAAGAGGAAA
F6155	CACTGGATGAGAGCGAATGA
F6890	CGGTATTTTATGCGAATGATGT
F7627	CCCAGCGTTCTGGAGGAAAT
F8256	AACGAGGGCAAACTTGGGAA
F8471	CATGGGTTTCATCCCCACGA
F8598	GTCGAGCCGCTCTGTATCAA
F8613	ATCAAGCGCATGGTCATGGA
R501	ACTGCGCGTCTAACATTCCA
R683	AACTCTGCTGTGTAGCGTCC
R793	TTGTTGCGTTGGTTCGGAAG
R930	GTGTTGGGTGCACACTTAGC
R1250	AACGGTGACAGCATTTGCAC
R2450	CCACAAGCTCCTTGTTGTGA
R2492	GAATCCAAAGACTGAAAACCC
R3435	CCCGTAACGGTATTCTGCAT
R3647	CTGCAGTCCAACTTCGATCA
R3700	GCTGCCCAAAAGTTGTAGCC
R3936	ACCCCCACCAAATAAGAAGG
R4515	TGCATTAAAGCTTGGGGTTC
R4807	CAGGTTTAGATGTACACGAGGATG
R4884	AACTTCGCAACCAACCAATC
R5453	TCGGGCACTGGACTACTTCT
R5871	ATTTCCCTCTCTCGCATCAA
R6256	CCACTGCCGTCACTAAACCT
R6300	TCATTCGCTCTCATCCAGTG
R6609	ATACTCCCCACTGCCATCAC
R6981	TCCTTAATGGCACGCACATA
R6995	TGCATTCCCACAATAGGTCTTT
R8500	TAGAATTCCAAGCCAGCGCA
R8863	TAAAATGCCATATATTGATATTAATCCA

## Supplementary Materials

The following are available online at http://www.mdpi.com/1999-4915/11/1/61/s1, Table S1: Primers used for virus detection in honey bee colonies.
