# Supplementary material for: The Phylogeny and Pathogenesis of Sacbrood Virus (SBV) Infection in European Honey Bees, Apis mellifera
Source: Viruses. 2019 Jan 14;11(1):61. doi: 10.3390/v11010061 (PMC6357158; doi:10.3390/v11010061)
Supplement: Supplementary file 1 [file viruses-11-00061-s001.pdf]

Supplementary Table S1: Primers used for virus detection in honey bee colonies.

Viruses	Primers (5’-3’)	Size	Reference
Acute bee paralysis virus	5’- accgacaaagggtatgatgc-3’<br>5’- cttgagtttgcggtgttcct-3’	123 bp	This study
Black queen cell virus	5’-ggagatgtatgcgctttatcgag-3’<br>5’-caccaaccgcataatagcgattg-3’	316 bp	Topley et al. “Detection of three honey bee viruses simultaneously by a single mutlplex reverse transcriptase PCR” African J. of Biotechnolgy. 4(7): 763-767. 2005).
Chromic bee paralysis virus	5’-agttgtcatggttaacaggatacgag-3’<br>5’-tctaatcttagcacgaaagccgag-3’	455 bp	Ribiere et al. 2002. Molecular diagnosis of chronic bee paralysis virus infection. Apidologie 33: 339-351
Deformed wing virus	5’- cgaaaccaacttctgaggaa -3<br>5′- gtgttgatccctgaggctta -3′	174 bp	This study
Kashmir bee virus	5'- gatgaacgtcgacctattga-3'<br>5'-tgtgggttggctatgagtca-3'	417 bp	Stoltz et al. 1995. Molecular diagnosis of Kashmir bee virus infection. J. Apic. Res. 34: 153-160.
Israeli acute paralysis virus	5’ –ctacaaggcgaatcacgct -3’<br>5’ –tttgcccactctacctaga- 3’	191 bp	This study
Sacbrood virus	5’-cggttgattggataatcagt-3’<br>5’-ctggactacttctcggtttgctg-3’	176 bp	This study
